# CHARACTERISTICS ASSOCIATED WITH FRAUD VICTIMIZATION AMONG OLDER CHINESE LIVING IN FOREST TOWN AREAS

**DOI:** 10.1093/geroni/igae098.1399

**Published:** 2024-12-31

**Authors:** Xiuting Cai, Guoyong Ma, Hongge Zhu

**Affiliations:** Northeast Forestry University, Harbin, Heilongjiang, China (People’s Republic); Northeast Forestry University, Harbin, Heilongjiang, China (People’s Republic); Northeast Forestry University, Harbin, Heilongjiang, China (People’s Republic)

## Abstract

The forest regions in northeast China, characterized by integrated industrial, urban, and rural communities known as forest town groups, have experienced significant demographic shifts over the past decade. These shifts included accelerated aging population combined with rapid population migration, making these areas emblematic of China’s modernization efforts in achieving urban-rural integration. Notably, the older population in these town groups has increasingly become targets of financial fraud. Against this backdrop, this study used panel data collected from 2012 to 2023 annually in these Chinese forest town areas. These surveys collected data on resident livelihoods and well-being, including educational attainment, income levels, living conditions, health status, and elder care services. This study focused on the roles of internet usage and financial literacy in relation to financial fraud victimization among older adults. Three major findings are as follows: 1) Internet usage correlates with a heightened risk of financial fraud, while financial literacy significantly reduces older adults’ susceptibility to fraud and financial losses. 2) Older women tend to have lower risks for financial fraud compared to their male counterparts, largely attributable to their higher financial literacy. 3) Other characteristics associated with higher financial fraud risks are loneliness, living alone, residing without children (empty-nest status) or living with disabilities. These findings emphasize the need for targeted interventions to improve financial literacy and internet safety practices among older adults in forest town areas, particularly focusing on the most vulnerable groups, to safeguard them against financial fraud.

